# Three new species and a new synonym of *Conocybe* section *Ochromarasmius* subsection *Dumetorae* (*Bolbitiaceae*, *Agaricales*) from northern China

**DOI:** 10.3897/mycokeys.132.184211

**Published:** 2026-05-19

**Authors:** Jia Tao, Yang Sheng, Jin-cheng Zhao, Zhi-ding Xiong, Song-ming Tang, Zong-long Luo, Han-bing Song

**Affiliations:** 1 College of Agriculture and Biological Science, Dali University, Dali 671003, Yunnan, China College of Agriculture and Biological Science, Dali University Dali China; 2 Co-Innovation Center for Cangshan Mountain and Erhai Lake Integrated Protection and Green Development of Yunnan Province, Dali University, Dali 671003, Yunnan, China Co-Innovation Center for Cangshan Mountain and Erhai Lake Integrated Protection and Green Development of Yunnan Province, Dali University Dali China

**Keywords:** Morphology, new synonym, new taxa, phylogeny, section *Ochromarasmius*

## Abstract

Based on morphological observations, this study reports the first discovery of species from section *Ochromarasmius* in China. Using combined internal transcribed spacer (ITS), nuclear large subunit ribosomal DNA (nrLSU), and translation elongation factor 1-alpha (*tef*1-α) sequences, Bayesian and maximum likelihood phylogenies were reconstructed for sect. *Ochromarasmius*. The resulting phylogenetic trees indicate that subsect. *Dumetorae* forms a monophyletic group. Subsect. *Subdumetorae*, in terms of both its definition and phylogenetic placement, is entirely nested within subsect. *Dumetorae*; therefore, it is treated as a later synonym. Furthermore, subsect. *Dumetorae* encompasses three new species from northern China: *Conocybe verrucosa*, *C. arenicola*, and *C. subfusispora*. Thus, the total number of species in subsect. *Dumetorae* is increased to 11 and that in sect. *Ochromarasmius* to 14. Additionally, based on morphological characteristics, putative phylogenetic positions are discussed for the other subsections and species within sect. *Ochromarasmius* that currently lack molecular sequence data. The study also provides an identification key for sect. *Ochromarasmius* subsect. *Dumetorae*, accompanied by morphological descriptions and line drawings of the new species.

## Introduction

*Conocybe* sect. *Ochromarasmius* (Singer) Hauskn. & Krisai was originally established as *Conocybe* subg. *Ochromarasmius* Singer for species characterized by rough-surfaced basidiospores (Singer 1947; Hausknecht 2009). In his original description, Singer defined the spore size as “less than 6 μm” but later expanded this definition after discovering *Conocybe radicata* Singer (Singer 1962). Subsequently, specialists in the family *Bolbitiaceae*, including Hausknecht, came to regard spore surface morphology as taxonomically less significant, leading to its reclassification as a section (Hausknecht and Krisai-Greilhuber 2006).

The classification of section *Ochromarasmius* at the subsection level was formalized in 2006. Based on differences in the type of pileipellis and the presence or absence of pleurocystidia and radicant structures, Hausknecht and Krisai-Greilhuber (2006) divided sect. *Ochromarasmius* into three subsections: subsect. *Juruensis* Hauskn. & Krisai, subsect. *Pleurocystidiatae* Hauskn. & Krisai, and subsect. *Dumetorae* Hauskn. & Krisai. Among these, subsect. *Juruensis* is monotypic, containing only the species *C. juruensis* (Henn.) Singer, which also serves as the type species of sect. *Ochromarasmius* (Hausknecht 2009). This subsection was established primarily based on the presence of abundant rostrate spheropedunculate cells in its pileipellis. Another monotypic subsection, subsect. *Pleurocystidiatae* includes only *C. radicata* Singer (Singer 1953), distinguished by its numerous lecythiform pleurocystidia and a radicant stipe base, which are the key diagnostic characteristics for this subsection (Hausknecht and Krisai-Greilhuber 2006).

In contrast, subsection *Dumetorae* is defined by the following features: the basidiocarp is mostly small and mycenoid in form, with a stipe base that is often slightly bulbous and never radicant. The spore print ranges from yellow-brown to rubiginous. Spores are tuberculous or rough; in one species, they appear smooth under light microscopy but are distinctly rough under scanning electron microscopy. Cheilocystidia are partly characterized by large capitula. Pleurocystidia and pseudoparaphyses are absent. The stipitipellis consists of lecythiform elements or a mixture of lecythiform, capilliform, clavate-cylindrical, or spherical elements. The pileipellis often bears lecythiform pileocystidia. This subsection is typically found in forest soil, meadows, or on rotting wood (Hausknecht and Krisai-Greilhuber 2006). In short, its main diagnostic features are the absence of both radicant structures and pleurocystidia. At the time of its delineation, subsect. *Dumetorae* included six species: *C. dumetorum* (Velen.) Svrček (including *C. dumetorum* var. *laricina* (Kühner) Hauskn. and *C. dumetorum* var. *phaeoleiospora* Hauskn.), *C. abjecta* (Berk. & Broome) Pegler, *C. spinulosa* Hauskn. & Krisai, *C. stictospora* Singer, *C. missionum* Singer, and *C. horakii* Watling & G.M. Taylor (Singer and Digilio 1951; Watling and Taylor 1987; Singer 1989; Hausknecht 1995; Hausknecht and Krisai-Greilhuber 1998; Hausknecht 2003).

In 2009, in his monograph of the genera *Conocybe* Fayod and *Pholiotina* Fayod in Europe, Hausknecht described the new species *C. subleiospora* Hauskn., which features a pseudorhiza but lacks pleurocystidia (Hausknecht 2009). Based on these characteristics, Hausknecht (2009) established a new subsection, subsect. *Subleiosporae* Hauskn., thereby increasing the number of subsections within sect. *Ochromarasmius* to four. In 2012, Malysheva described another new species, *C. mandshurica* E.F. Malysheva. This species is characterized by tuberculate-roughened basidiospores and a pileipellis containing hair-like elements. Based on these features, Malysheva established a further new subsection, subsect. *Subdumetorae* E.F. Malysheva, with *C. mandshurica* as its type species, bringing the total number of subsections in the section to five (Malysheva 2012a).

Five years later, Malysheva described two additional species in section *Ochromarasmius* based on materials from Russia: *Conocybe confundens* E.F. Malysheva and *C. incerta* E.F. Malysheva (Malysheva 2017). However, neither was assigned to any of the existing subsections. Currently, as of April 2026, sect. *Ochromarasmius* includes a total of 12 species.

Species of section *Ochromarasmius* are widely distributed globally across habitats such as grasslands, forest soils, and decaying wood. However, prior to this study, no members of this section had been reported from China, highlighting a knowledge gap that motivated the present research. China’s vast territory encompasses a rich variety of ecosystems, spanning continuous climatic zones from tropical to cold temperate, which provides suitable conditions for the survival and differentiation of diverse fungi and suggests a high likelihood of discovering species of sect. *Ochromarasmius* within the country. Given this potential, field surveys conducted in northern China between 2023 and 2026 successfully collected specimens belonging to this section. The identification and documentation of these species not only provide the first record of sect. *Ochromarasmius* in China but also significantly expand its known geographic distribution.

## Materials and methods

### Abbreviations

For Latin names: ***B***. = *Bolbitius*; ***C***. = *Conocybe*; ***Ca***. = *Candolleomyces*; ***Co***. = *Conocybula*; ***Con***. = *Conobolbitina*; ***D***. = *Descolea*; ***G***. = *Galerella*; ***N***. = *Nothofagus*; ***P***. = *Pholiotina*; ***Ps***. = *Psathyrella*.

### Sampling and morphological analyses

The specimens used in this study were collected in Jilin Province and Gansu Province, China, between 2023 and 2025. Specimen collection and morphological examinations were conducted following the standardized protocols described by Song and Bau (2025). Fresh basidiomata were collected, photographed *in situ*, and documented with ecological data (habitat, substrate, and coordinates). Specimens were rapidly dried using silica gel for preservation and are deposited in the Fungarium of Jilin Agricultural University (HMJAU/FJAU).

For microscopic analysis, rehydrated tissue sections were examined in distilled water, 5% KOH, and sometimes 1% Congo red. A 25% ammonia solution was used to test for reactions on lamellae (Hausknecht 2009). Observations and photomicrography were performed using a Nikon Eclipse 80i microscope. For scanning electron microscopy (SEM), dried lamellar fragments were sputter-coated with gold and examined with a Hitachi TM4000 Plus II tabletop microscope.

Macroscopic colors were described with reference to the RAL color system. Microscopic measurements (e.g., of basidiospores, basidia, and cystidia) followed the statistical format detailed in Song and Bau (2025), where measurements are presented as (*a*)*b*–*c*(*d*) and the notation (*n*/*m*/*p*) indicates the number of spores measured from a specified number of basidiomata and collections.

### DNA extraction, PCR amplification, and sequencing

Laboratory procedures for DNA extraction, PCR amplification, and sequencing were performed following the protocol detailed in Song and Bau (2025). In summary, total genomic DNA was extracted using the same method. The ITS region was amplified with primers ITS1F/ITS4 (White et al. 1990; Gardes and Bruns 1993), nrLSU with LR0R/LR7 (Vilgalys and Hester 1990; Moncalvo et al. 2000), and the *tef*1-α gene with EF1-983F/EF1-2218R (Rehner and Buckley 2005), using the same reaction mixtures and thermal cycling conditions. PCR was performed on a Bio-Rad T100™ Thermal Cycler.

PCR products were purified after verification on 1% agarose gels and sent to Sangon Biotech (Shanghai) Co., Ltd. for sequencing. The ITS region was sequenced in one direction, whereas nrLSU and *tef*1-α were sequenced in both directions. Raw sequence chromatograms were edited in BioEdit v7.2.5 (Hall 1999), with specific trimming criteria applied: for ITS, the region from the 3' end of 18S to the 5' end of 28S was selected; for nrLSU and *tef*1-α, low-quality ends were trimmed. Polymorphic sites were encoded using IUPAC ambiguity codes. Sequence identity was confirmed via BLAST searches against the NCBI database. The final sequences were deposited in GenBank (see accession numbers in Table 1).

**Table 1.** Information on the DNA sequences used to reconstruct phylogenetic trees. Newly generated sequences are emphasized in bold; “–” indicates missing sequences. T = holotype.

Taxon	Voucher specimen	GenBank accession numbers			Origin	References
		ITS	nrLSU	*tef*1-α		
*Bolbitius coprophilus*	HMJAU64958	OQ780315	OQ758216	–	China	Song and Bau 2023
*B. coprophilus*	SZMC-NL-2640	JX968253	JX968370	–	Hungary	Tóth et al. 2013
*B. reticulatus*	WU30001	JX968249	JX968366	JX968455	Hungary	Tóth et al. 2013
*B. subvolvatus*	WU28379	JX968248	JX968365	JX968454	Italy	Tóth et al. 2013
*Candolleomyces leucotephrus*	SZMC-NL-1953	FM163226	FM160683	FM897219	Hungary	Nagy et al. 2011
*Conocybe alboradicans*	SZMC-NL-3226	JX968219	JX968336	JX968435	Hungary	Tóth et al. 2013
*C. alkovii*	FJAU71714	PX589399	PX589415	PX610261	China	Song et al. 2026
*C. anthracophila*	WU14367	JX968212	JX968329	JX968430	Italy	Tóth et al. 2013
*C. antipus*	WU19791	JX968215	JX968332	JX968432	Austria	Tóth et al. 2013
***C. arenicola***	**FJAU78830 T**	**PX746924**	**PX746932**	**PX754428**	**China**	**This study**
***C. arenicola***	**FJAU78833**	**PX746925**	**PX746933**	**PX754429**	**China**	**This study**
*C. bispora*	SZMC-NL-2573	JX968203	JX968320	JX968423	Hungary	Tóth et al. 2013
***C. brachypodii***	**HMJAU64927**	**PX746930**	**PX746938**	**PX754434**	**China**	**This study**
*C. ceracea*	HMJAU64951	OQ758110	OQ758218	OQ758305	China	Song and Bau 2023
*C. cettoiana*	WU10436	JX968218	JX968335	–	Italy	Tóth et al. 2013
*C. confundens*	LE313077 T	KY614063	–	–	Russia	Malysheva 2017
*C. coniferarum*	FJAU72632	PX589398	PX589414	PX610260	China	Song et al. 2026
*C. crispella*	WU27367	JX968208	JX968325	JX968426	Australia	Tóth et al. 2013
*C. cylindrospora*	HMJAU42440 T	MG250375	OQ758203	PX610262	China	Liu and Bau 2018; Song and Bau 2023
*C. deliquescens*	HMJAU61998	OP373403	OQ758204	OQ758292	China	Song and Bau 2023
*C. dumetorum*	SZMC-NL-2693	JX968201	JX968318	JX968421	Sweden	Tóth et al. 2013
*C. enderlei*	WU21272	JX968163	JX968279	–	Italy	Tóth et al. 2013
*C. gigasperma*	SZMC-NL-3972	JX968179	JX968295	JX968403	Slovakia	Tóth et al. 2013
*C. graminis*	WU13466	JX968195	JX968311	–	Austria	Tóth et al. 2013
*C. hausknechtii*	LE253789 T	JQ247194	–	–	Russia	Malysheva 2012b
*C. herbarum*	WU22193	JX968193	JX968309	–	Austria	Tóth et al. 2013
*C. hornana*	SZMC-NL-3499	JX968178	JX968294	JX968402	Slovakia	Tóth et al. 2013
*C. incerta*	LE313017 T	KY614062	–	–	Russia	Malysheva 2017
*C. intrusa*	WU25546	JX968211	JX968328	JX968429	Finland	Tóth et al. 2013
*C. inocybeoides*	SZMC-NL-3589	JX968202	JX968319	JX968422	Hungary	Tóth et al. 2013
*C. karinae*	WU28526	JX968151	JX968268	JX968384	Germany	Tóth et al. 2013
*C. leporina*	SZMC-NL-2380	JX968177	JX968293	JX968401	Hungary	Tóth et al. 2013
*C. macrospora*	WU17030	JX968175	JX968291	–	Germany	Tóth et al. 2013
*C. mandshurica*	LE262844 T	JQ247197	–	–	Russia	Malysheva 2012a
***C. mesospora***	**FJAU72663**	**PX746931**	**PX746939**	**PX754435**	**China**	**This study**
*C. monikae*	WU22612	JX968200	JX968317	JX968420	Austria	Tóth et al. 2013
*C. olivaceopileata*	LE313106 T	KY614059	–	–	Russia	Malysheva 2017
*C. parapilosella*	JLS3063 T	MN872706	–	–	Spain	Siquier and Salom 2020
*C. pilosella*	HMJAU64957	OQ780306	OQ758206	OQ758295	China	Song and Bau 2023
*C. praticola*	HMJAU64965	OQ780303	PX589412	PX610258	China	Song and Bau 2023; Song et al. 2026
*C. pseudocrispa*	HMJAU64944	OQ780308	OQ758211	OQ758298	China	Song and Bau 2023
*C. pubescens*	WU20759	JX968170	JX968286	JX968396	Italy	Tóth et al. 2013
*C. punjabensis*	Skp066 T	MH981969	–	–	Pakistan	Izhar et al. 2019
*C. romagnesii*	WU26605	JX968206	JX968323	JX968424	Italy	Tóth et al. 2013
*C. rostellata*	SZMC-NL-2499	JX968162	JX968278	JX968390	Sweden	Tóth et al. 2013
*C. rufostipes*	HMJAU64937	OQ758120	OQ758227	OQ758317	China	Song and Bau 2023
*C. semiglobata*	FJAU72755	PX589397	PX589413	PX610259	China	Song et al. 2026
*C. siennophylla*	HMJAU64966	OQ780312	OQ758210	OQ758297	China	Song and Bau 2023
*C. singeriana*	WU22129	JX968166	JX968282	JX968393	Austria	Tóth et al. 2013
*C. solitaria*	WU20903	JX968214	JX968331	JX968431	India	Tóth et al. 2013
***C. subfusispora***	**FJAU78834 T**	**PX746927**	**PX746935**	**PX754431**	**China**	**This study**
***C. subfusispora***	**FJAU78835**	**PX746926**	**PX746934**	**PX754430**	**China**	**This study**
*C. tenera*	SZMC-NL-1615	JX968180	JX968296	JX968404	Hungary	Tóth et al. 2013
*C. tuxlaensis*	SZMC-NL-1897	JX968164	JX968280	JX968391	Hungary	Tóth et al. 2013
*C. vaginata*	WU25703	JX968204	JX968321	–	Sri Lanka	Tóth et al. 2013
***C. verrucosa***	**FJAU78839 T**	**PX746929**	**PX746937**	**PX754433**	**China**	**This study**
***C. verrucosa***	**FJAU78841**	**PX746928**	**PX746936**	**PX754432**	**China**	**This study**
*C. volvicystidiata*	LIP0001212 T	KY346827	–	–	France	Hausknecht and Broussal 2016
*C. watlingii*	WU22744	JX968172	JX968288	JX968398	Finland	Tóth et al. 2013
*C. zuccherellii*	WU12421	JX968213	JX968330	–	Italy	Tóth et al. 2013
*Conocybula. coprophila*	HMJAU62008	OR995662	OR995712	PP000855	China	Song and Bau 2024
*Co. cyanopus*	HMJAU62007	OR995663	OR995713	PP000856	China	Song and Bau 2024
*Co. smithii*	HMJAU62001	OP373407	OQ758215	OQ758300	China	Song and Bau 2023; Song and Bau 2024
*Conobolbitina aeruginosa*	WU27104	JX968247	JX968364	–	Germany	Tóth et al. 2013; Song and Bau 2024
*Con. dasypus*	HMJAU62002	OR995661	OR995711	PP000854	China	Song and Bau 2024
*Con. lignicola*	FJAU72922 T	PX612217	PX612214	PX610264	China	Song et al. 2026
*Con. pygmaeoaffinis*	WU16600	JX968149	JX968382	–	Austria	Tóth et al. 2013; Song and Bau 2024
*Descolea antarctica*	NZ5182	AF325647	–	–	USA	Peintner et al. 2001
*D. quercina*	HMJAU64959	OQ780313	OQ758213	OQ758299	China	Song and Bau 2023
*Galerella nigeriensis*	CNF1/5859 T	JX968251	JX968368	JX968457	Nigeria	Tóth et al. 2013
*G. plicatella*	GC-07468	OQ845969	OR039001	–	Italy	Direct Submission
*Pholiotina dentatomarginata*	SZMC-NL-2921	JX968258	JX968374	JX968460	Hungary	Tóth et al. 2013
*P. eburnea*	HMJAU65035 T	OR995694	OR994097	PP000886	China	Song and Bau 2024
*P. intermedia*	HMJAU62014	OR995667	OR995717	PP000860	China	Song and Bau 2024
*P. serrata*	HMJAU62006	OP538570	OQ758217	OQ758301	China	Song and Bau 2023
*P. sulciceps*	HMJAU65100 T	OR995710	OR994113	PP000902	China	Song and Bau 2024
*P. vexans*	HMJAU45078	OR995669	OR995719	PP000862	China	Song and Bau 2024
*Psathyrella piluliformis*	HMJAU37922	MG734716	MW413364	MW411001	China	Yan and Bau 2018

### Phylogenetic analyses

Phylogenetic analyses were conducted following the pipeline of Song and Bau (2025). Briefly, sequence data were obtained from GenBank (Table 1) and supplemented with the newly generated sequences. The ITS, nrLSU, and *tef*1-α loci were aligned using the online MAFFT tool (Katoh et al. 2019), manually adjusted in MEGA7 (Kumar et al. 2016), and concatenated in PhyloSuite v2 (Zhao et al. 2025). The best-fit partitioning scheme and substitution models were selected with ModelFinder v2.2.0 (Kalyaanamoorthy et al. 2017). Maximum likelihood analysis was performed using IQ-TREE 3 (Nguyen et al. 2015; Wong et al. 2025) with 1,000 standard bootstrap replicates, and Bayesian inference was run using MrBayes v3.2.7a (Ronquist et al. 2012) for 1,000,000 generations, until the average standard deviation of split frequencies had fallen below 0.01 (the final value was 0.006). Consensus trees were visualized in iTOL (Letunic and Bork 2019) and finalized with Adobe graphics software. *Psathyrella* and *Candolleomyces* (Song and Bau 2023) were selected as the outgroup.

## Results

### Phylogenetic analyses

**Figure 1.**
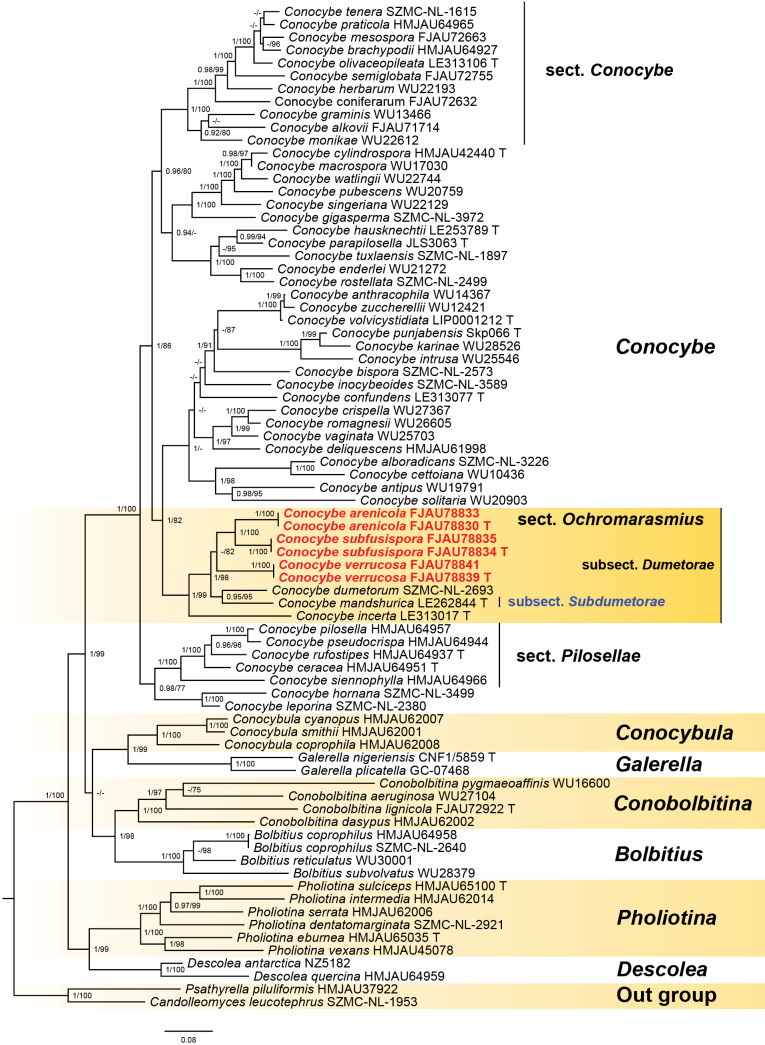
The phylogenetic relationships of *Conocybe* sect. *Ochromarasmius* within the family *Bolbitiaceae* were analyzed using Bayesian inference and maximum likelihood methods based on a multi-marker dataset (ITS, nrLSU, and *tef*1-α). In the resulting phylogenetic tree, the newly proposed species names are highlighted in bold red, whereas the new synonym subsection is indicated in bold blue. T = holotype.

The phylogenetic tree was reconstructed using Bayesian inference based on a combined dataset of ITS, nrLSU, and *tef*1-α sequences. The maximum likelihood (ML) tree is omitted from the figure because its topology was nearly identical to the Bayesian tree, with the exception of *C. inocybeoides* SZMC-NL-3589 and *C. confundens* LE313077, which occupied unstable positions with low node support; however, both still clustered within the sister clade of sect. *Ochromarasmius* in the ML phylogeny. Bayesian posterior probabilities (PP ≥ 0.9) and ML bootstrap values (MLbs ≥ 70%) are shown on the nodes; nodes with values below these thresholds are indicated by “–” (Fig. 1).

The multi-marker dataset (ITS + nrLSU + *tef*1-α) had aligned lengths of 849 bp for ITS, 1,365 bp for nrLSU, and 1,108 bp for *tef*1-α. The final concatenated alignment contained 78 sequences comprising 3,252 sites, including 1,496 distinct patterns, 1,084 parsimony-informative sites, 263 singleton sites, and 1,905 constant sites. For the ML analysis, the best-fit substitution models selected under the Akaike information criterion (AIC) were GTR+F+I+R4 for ITS, GTR+F+I+R3 for nrLSU, and GTR+R6 for *tef*1-α. For the Bayesian analysis, the best-fit models selected under the Bayesian information criterion (BIC) were GTR+F+I+G4 for ITS, HKY+I+G4 for nrLSU, and SYM+I+G4 for *tef*1-α.

In the phylogenetic tree (Fig. 1), new species are highlighted in bold red, whereas the newly proposed synonym is indicated in bold blue. *Ps. piluliformis* (Bull.) P.D. Orton and *Ca. leucotephrus* (Berk. & Broome) D. Wächt. & A. Melzer were designated as outgroups. Sect. *Ochromarasmius* forms a distinct, strongly supported monophyletic clade (PP/MLbs = 1/82), within which the phylogenetic placement of subsect. *Dumetorae* is clarified. Sequences for the remaining subsections, including that of the type species for sect. *Ochromarasmius* are currently unavailable.

Within subsection *Dumetorae* of *Conocybe* sect. *Ochromarasmius*, specimens FJAU78839 and FJAU78841 form a distinct clade with maximum support (1/100). This clade is sister to a second clade comprising specimens FJAU78834, FJAU78835, FJAU78830, and FJAU78833, although this sister relationship receives only moderate support (–/82). The moderate support for this node may be due to undiscovered transitional lineages between the two groups. Within the latter clade, specimens FJAU78830 and FJAU78833 form a maximally supported subclade (1/100), which is sister to the equally maximally supported subclade formed by FJAU78834 and FJAU78835 (1/100).

A BLAST search of the ITS sequence from specimen FJAU78839 against the NCBI database showed similarity percentages of 85.96% to *C. dumetorum* (SZMC-NL-2693), 85.76% to *C. mandshurica* (LE262844), 85.32% to FJAU78834, 83.07% to *C. incerta* (LE313017), and 82.85% to FJAU78830. For specimen FJAU78834, the ITS sequence showed 87.69% similarity to FJAU78830, 85.76% to *C. mandshurica* (LE262844), 84.57% to *C. dumetorum* (SZMC-NL-2693), and 82.63% to *C. incerta* (LE313017). The ITS sequence of FJAU78830 exhibited 84.74% similarity to *C. mandshurica* (LE262844), 82.82% to *C. incerta* (LE313017), and 82.63% to *C. dumetorum* (SZMC-NL-2693).

Accordingly, based on morphological evidence and their distinct phylogenetic positions, three new species are proposed: *C. verrucosa* (represented by specimens FJAU78839 and FJAU78841), *C. arenicola* (FJAU78830 and FJAU78833), and *C. subfusispora* (FJAU78834 and FJAU78835). Additionally, subsect. *Subdumetorae* is treated as a new synonym of subsect. *Dumetorae*.

### Taxonomy

#### Conocybe

Taxon classification

Fungi

AgaricalesBolbitiaceae

sect. Ochromarasmius (Singer) Hauskn. & Krisai, Österr. Z. Pilzk. 15: 207 (2006)

EA054DDC-B486-5610-B995-1EDB126D7C70

##### Type species.

*Conocybe juruensis* (Henn.) Singer.

##### Basionym.

*Conocybe* subgen. *Ochromarasmius* Singer, Mycologia 39: 88 (1947).

##### Description.

As in Hausknecht and Krisai-Greilhuber (2006).

##### Notes.

Amid the taxonomic controversy regarding the valid generic name (*Conocybe* vs. *Galera* (Fr.) P. Kumm.), Singer, following Fayod, adopted *Conocybe*. Based on basidiospore ornamentation, Singer divided *Conocybe* into two subgenera: subgen. *Euconocybe*, characterized by smooth basidiospores, and subgen. *Ochromarasmius* Singer, distinguished by faintly tuberculate-roughened basidiospores (Fries 1821; Fayod 1889; Singer 1947). Moreover, the present study reports the first discovery of taxa belonging to sect. *Ochromarasmius* in China.

#### Conocybe

Taxon classification

Fungi

AgaricalesBolbitiaceae

subsect. Dumetorae Hauskn. & Krisai, Österr. Z. Pilzk. 15: 208 (2006)

7EA91855-8729-5721-B104-48A46BE8183B

##### Type species.

*Conocybe dumetorum* (Velen.) Svrček.

##### Synonymy.

*Conocybe* subsect. *Subdumetorae* E.F. Malysheva, Mikol. Fitopatol. 46(4): 241 (2012).

##### Description.

As in Hausknecht and Krisai-Greilhuber (2006).

##### Notes.

Based on morphological and phylogenetic evidence, subsect. *Subdumetorae* is treated as a synonym of subsect. *Dumetorae*. The rationale for this taxonomic revision is provided in the Discussion.

#### Conocybe verrucosa

Taxon classification

Fungi

AgaricalesBolbitiaceae

H.B. Song & J. Tao sp. nov.

CED0B1D2-5730-5E8B-A226-B62B07128477

861722

**Figure 2.**
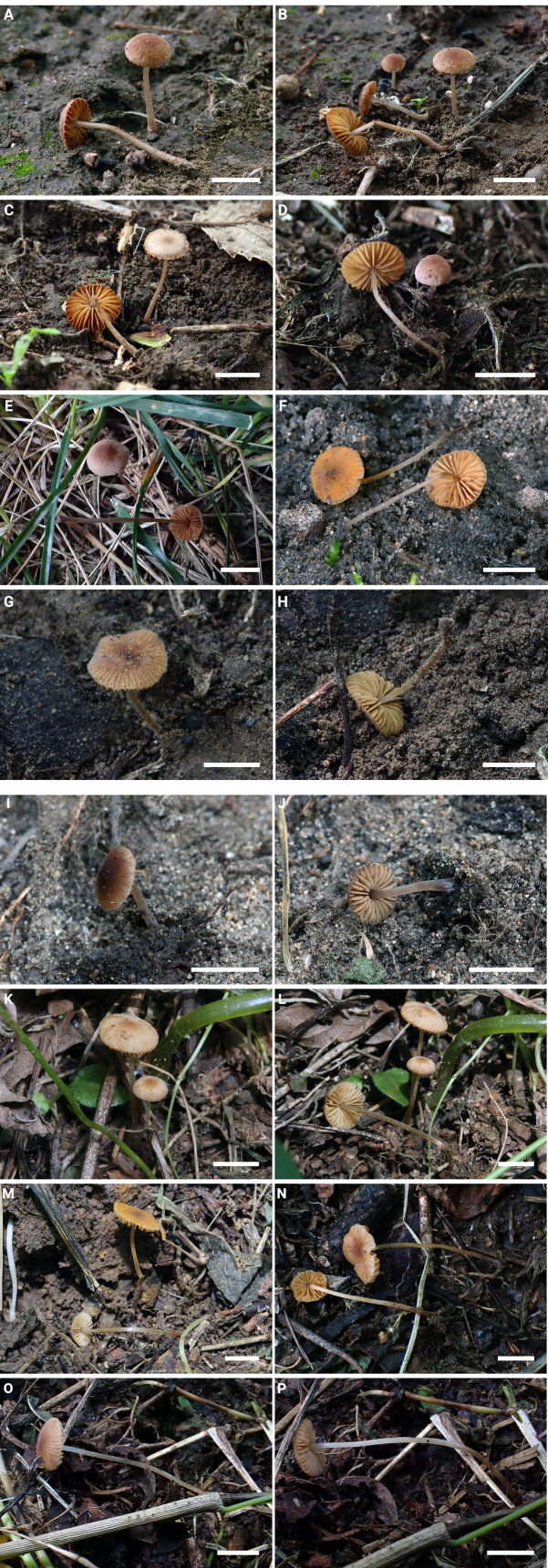
Basidiomata of *Conocybe* sect. *Ochromarasmius* species. **A**. *C. verrucosa* (FJAU78839 T); **B**. *C. verrucosa* (FJAU78840); **C**. *C. verrucosa* (FJAU78841); **D**. *C. verrucosa* (FJAU78843); **E**. *C. verrucosa* (FJAU78845); **F**. *C. arenicola* (FJAU78830 T); **G, H**. *C. arenicola* (FJAU78832); **I, J**. *C. arenicola* (FJAU78831); **K, L**. *C. subfusispora* (FJAU78834 T); **M**. *C. subfusispora* (FJAU78838); **N**. *C. subfusispora* (FJAU78836); **O, P**. *C. subfusispora* (FJAU78835). Scale bars: 0.5 cm, T = holotype.

**Figure 3.**
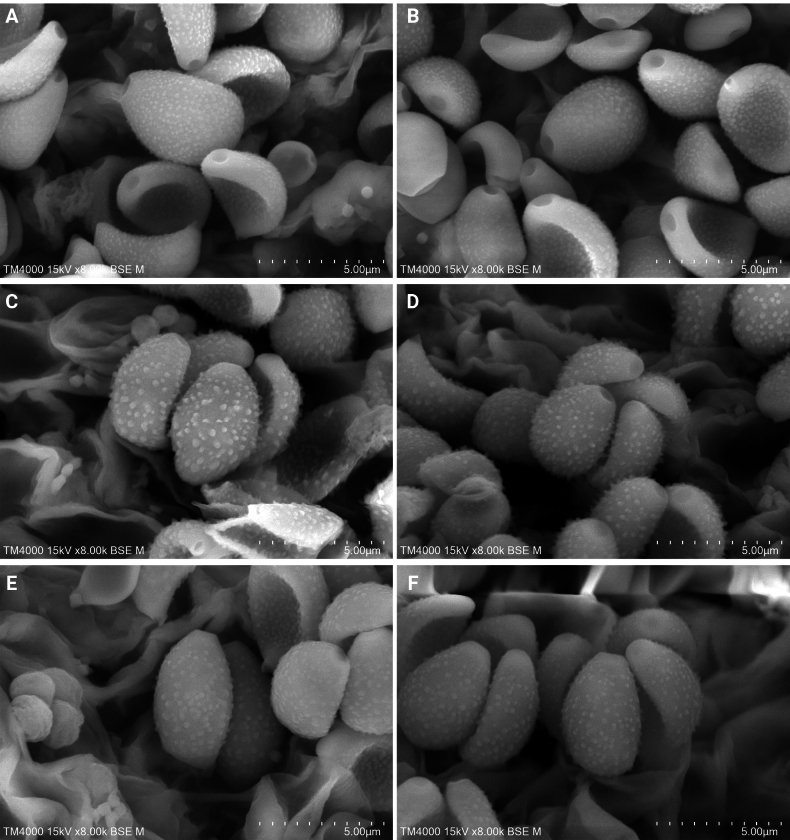
Basidiospores under electron microscopy of *Conocybe* sect. *Ochromarasmius* species. **A, B**. *C. verrucosa* (FJAU78839 T); **C, D**. *C. arenicola* (FJAU78830 T); **E, F**. *C. subfusispora* (FJAU78834 T). Scale bars: 5 μm, T = holotype.

**Figure 4.**
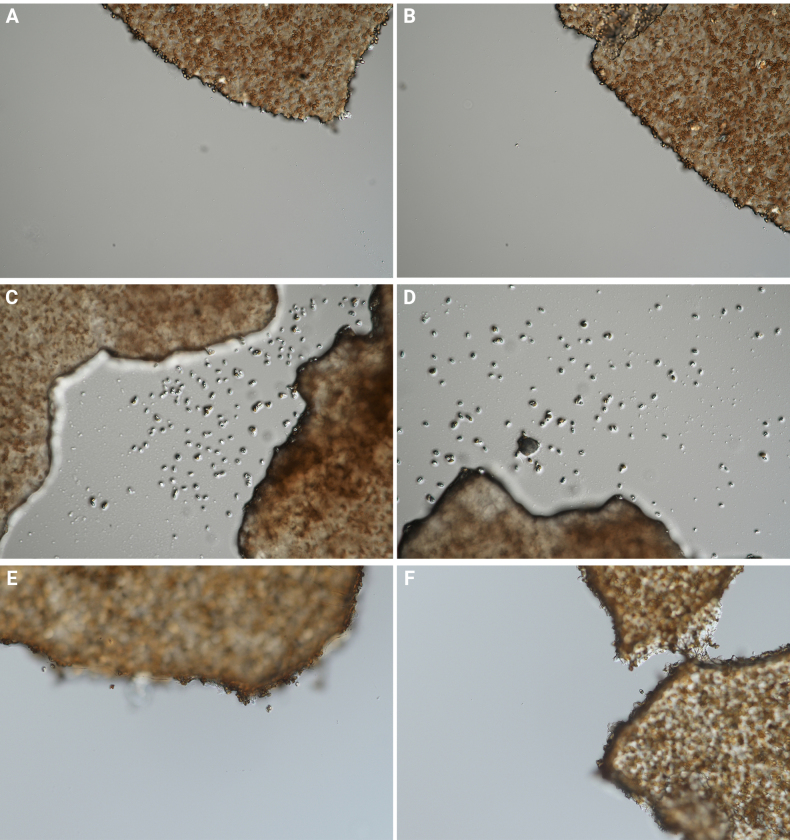
Ammonia reaction illustration of species in *Conocybe* sect. *Ochromarasmius*. **A, B**. *C. verrucosa* (FJAU78839 T); **C, D**. *C. arenicola* (FJAU78830 T); **E, F**. *C. subfusispora* (FJAU78834 T). T = holotype.

**Figure 5.**
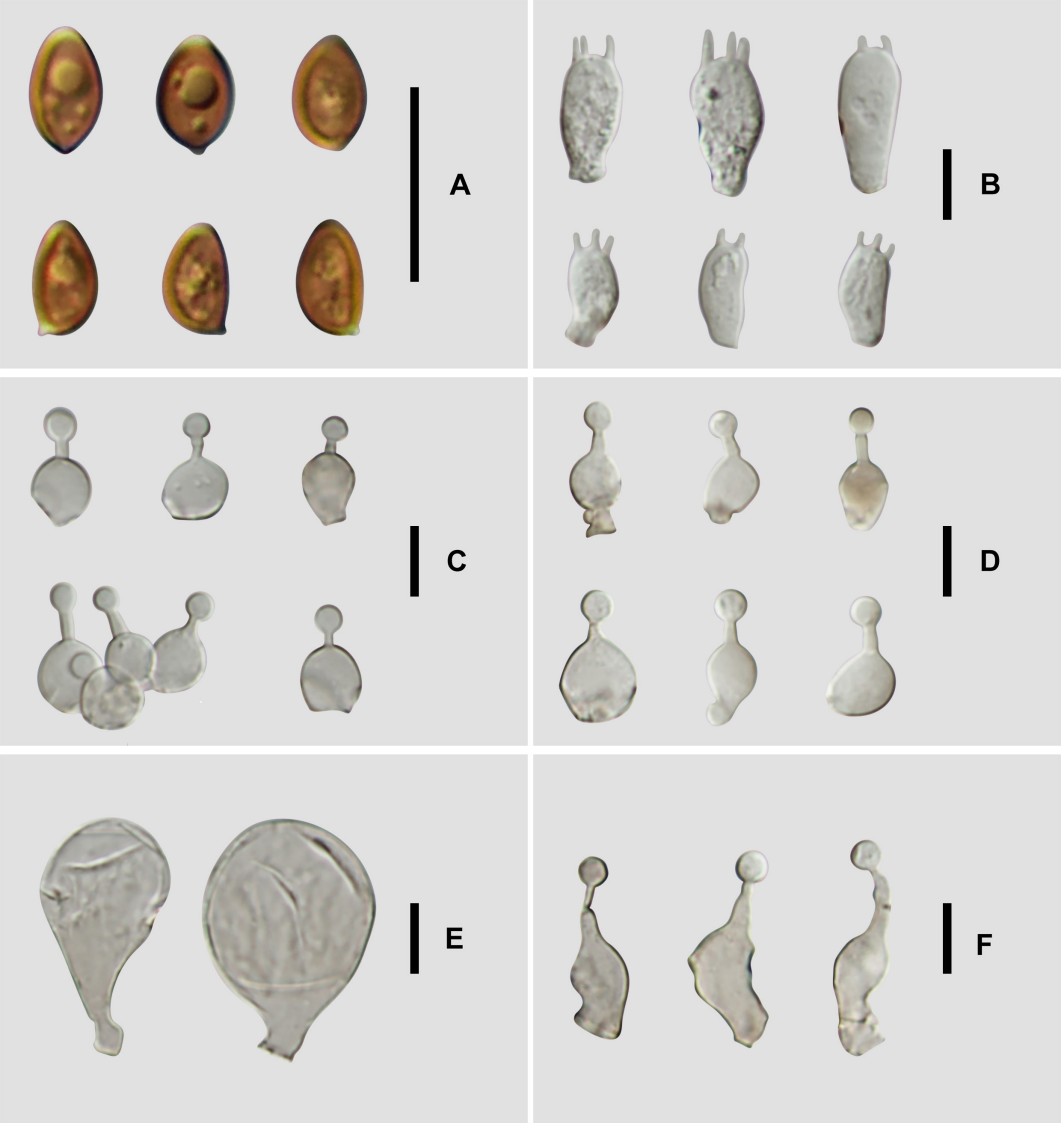
Microscopic structure images of *C. verrucosa* (FJAU78839). **A**. Basidiospores; **B**. Basidia; **C**. Cheilocystidia; **D**. Caulocystidia; **E**. Pileipellis; **F**. Pileocystidia. Scale bars: 10 μm (**A–F**).

**Figure 6.**
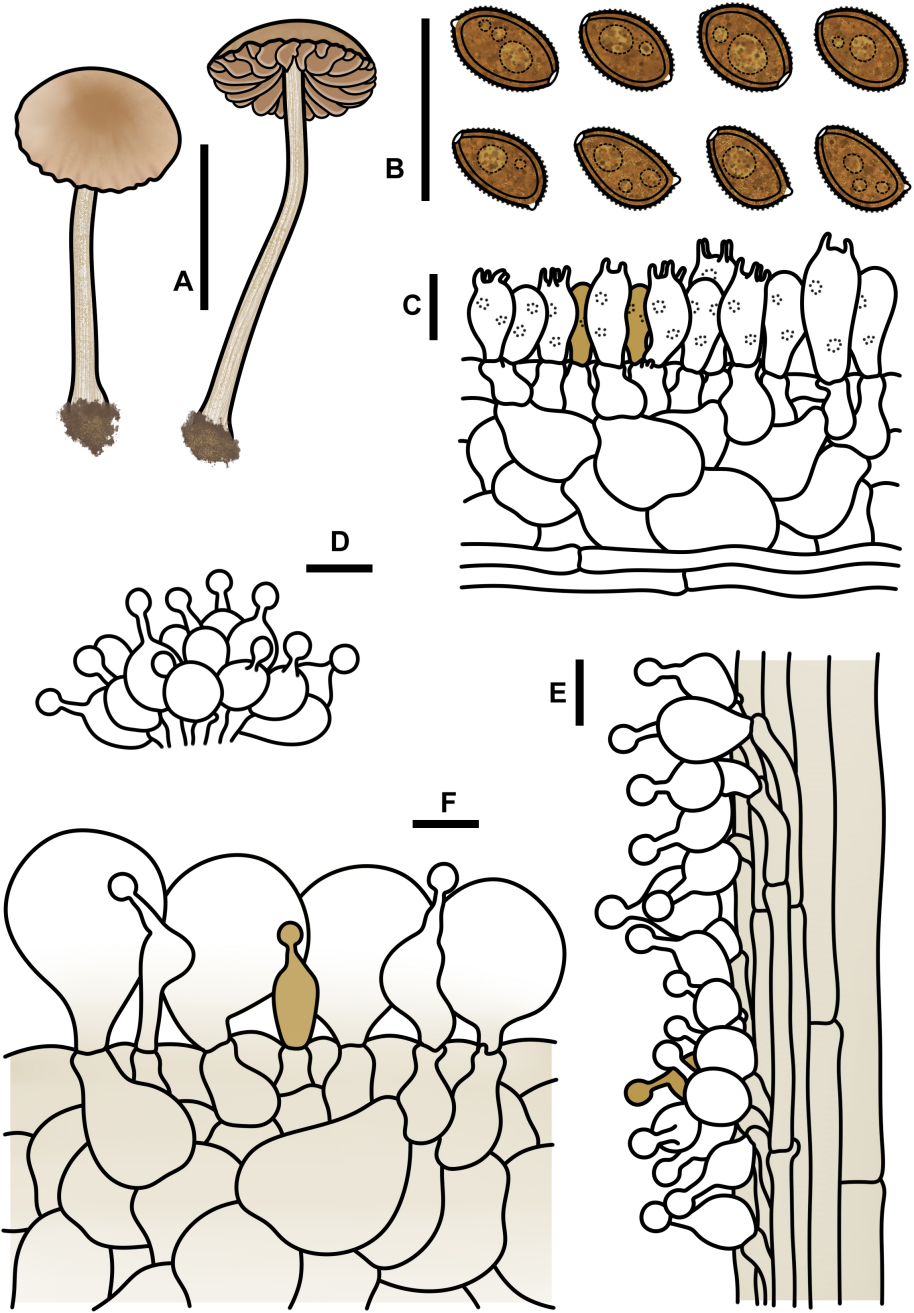
*Conocybe verrucosa* (FJAU78839). **A**. Basidiomata; **B**. Basidiospores in KOH; **C**. Hymenium and subhymenium; **D**. Cheilocystidia; **E**. Stipitipellis; **F**. Pileipellis; Scale bars: 0.5 cm (**A**); 10 μm (**B–F**).

Figs 2A–E, 3A, 3B, 4A, 4B, 5, 6

##### Etymology.

“verrucosa” refers to basidiospores having ornamentation on their surface, characterized by wart-like protrusions.

##### Holotypus.

China • Jilin Province, Jilin City, Songhua River bank, 20 July 2023, 43°50'17"N, 126°33'38"E, alt. 196 m, Han-bing Song, S23072004 (FJAU78839).

##### Diagnosis.

The species is primarily characterized by a glabrous pileus, basidiospores slightly lentiform, germ pore lacking a papilla, lecythiform cheilocystidia, caulocystidia, and pileocystidia, and a pileipellis devoid of hair-like elements. This combination of features distinguishes it from other species within *Conocybe* sect. *Ochromarasmius*.

##### Description.

Basidiomata mycenoid. Pileus diameter 0.3–1.5 cm, initially hemispherical to convex, later plano-convex to applanate, margin straight, undulate. Moist pileus center signal brown (RAL 8002) to copper brown (RAL 8004) or clay brown (RAL 8003), margin mottled light ivory (RAL 1015) to ivory (RAL 1014). Slightly dried pileus center signal brown (RAL 8002) to clay brown (RAL 8003), margin ivory (RAL 1014) to beige red (RAL 3012). Hygrophanous, smooth, glabrous, striations indistinct. Context thin, color same as pileus, odor absent, taste absent. Lamellae adnexed to narrowly adnate, ventricose, distant, unequal length, ochre brown (RAL 8001) to copper brown (RAL 8004), margin even to slightly eroded. Stipe length 1–2.5 cm, diameter 0.5–1 mm, cylindrical, slightly transparent, sand yellow (RAL 1002) to green brown (RAL 8000) or clay brown (RAL 8003), surface pruinose, longitudinally fibrillose-striate, base subbulbous.

##### Basidiospores.

(60/3/3) (5.3–)5.4–6.8(–6.9) × (3.1–)3.2–4.1(–4.2) μm, *Q* = (1.45–)1.5–1.79(–1.88), *Qm* = 1.63(±0.09), slightly lentiform, ellipsoid to oblong, frontal view nearly ovoid, lateral view slightly amygdaliform, wall thick, ornamentation warty to pointed, faintly tuberculate-roughened, containing oil droplets, germ pore approximately 1 μm diameter, in KOH clay brown (RAL 8003) to fawn brown (RAL 8007). Basidia (10–)11–21 × (5–)6–10(–11) μm, clavate, 4-spored, occasionally 2-spored, sterigmata 2–5 μm long, vacuolar inclusions present. Cheilocystidia (14–)15–20 × 7–10 μm, lecythiform, capitula 3–5 μm wide. Pleurocystidia absent. Caulocystidia 15–22(–26) × (5–)7–10(–11) μm, lecythiform, capitula 3–6 μm wide. Pileipellis epithelioid hymeniderm, cells (23–)24–45(–49) × 17–28(–32) μm, spheropedunculate, base with brown beige (RAL 1011) pigment. Pileocystidia (19–)20–30(–35) × (6–)7–12 μm, lecythiform, capitula 3–5 μm wide, some pigment-filled. Clamp connections present on all structures. Reaction with ammonia solution negative.

##### Habitat.

Grows scattered on grassy ground under elm trees in summer.

##### Known distribution.

Jilin Province, Gansu Province, China.

##### Additional specimens measured.

China • Jilin Province, Jilin City, Songhua River bank, 20 July 2023, 43°50'17"N, 126°33'38"E, alt. 196 m, Han-bing Song, S23072002 (FJAU78840), S23072003 (FJAU78841), S23072007 (FJAU78842); China • Jilin City, Songhua River bank, 20 July 2023, 43°50'16"N, 126°33'39"E, alt. 200 m, Han-bing Song, S23072004 (FJAU78843), S23072005 (FJAU78844); China • Gansu Province, Zhangye City, Hexi University, 5 August 2023, 38°56'53"N, 100°26'23"E, alt. 1619 m, Hong Chen, C2380510 (FJAU78845).

##### Notes.

Within subsection *Dumetorae*, this species can be distinguished from *Conocybe dumetorum* and *C. dumetorum* var. *laricina* by the latter two having a pruinose-pubescent, distinctly striate pileus, non-lentiform basidiospores, and a pileipellis with scattered hairs (Hausknecht 1995). Although this species and *C. dumetorum* var. *haeoleiospora* both share a glabrous pileus, the latter differs in its dark brown to nearly black-brown pileus color, pronounced striations, and non-lentiform basidiospores (Hausknecht 1995). Compared with *C. abjecta*, which has slightly larger basidiospores (up to 8.5 μm long), lecythiform and non-lecythiform caulocystidia, and lacks pileocystidia, the present species is distinct (Hausknecht 2003). It differs from *C. spinulosa*, which has an umbonate pileus, non-lecythiform caulocystidia, and occurs on rotten hardwood in tropical rainforests (Hausknecht and Krisai-Greilhuber 1998). Separation from *C. stictospora* is based on the latter possessing mixed lecythiform and non-lecythiform caulocystidia, lacking pileocystidia, and inhabiting decomposing leaves in tropical forests (Singer 1989). This species is distinguishable from *C. missionum*, which has slightly larger, somewhat angular basidiospores with a distinct, papillate germ pore and a type locality in Argentina (Singer and Digilio 1951). It is distinguished from *C. horakii* by the latter having mixed lecythiform and non-lecythiform caulocystidia and occurring on wood of *Nothofagus fusca* (Hook.f.) Oerst. in New Zealand (Watling and Taylor 1987). This species differs from *C. mandshurica*, which has an obtusely conical to conical-convex, hairy pileus, larger basidiospores, and a pileipellis with scattered hairs (Malysheva 2012a). It also differs from *C. incerta*, characterized by a subumbonate, hairy, and distinctly striate pileus and cystidia with a larger capitulum diameter (Malysheva 2017). Phylogenetically, this species forms an independent clade sister to the clade containing *C. arenicola* and *C. subfusispora*. The latter two species are distinguished by their distinctly pubescent pileus.

#### Conocybe arenicola

Taxon classification

Fungi

AgaricalesBolbitiaceae

H.B. Song & S.M. Tang sp. nov.

691DD6AD-9905-5E8D-9811-550B6665CD44

861723

**Figure 7.**
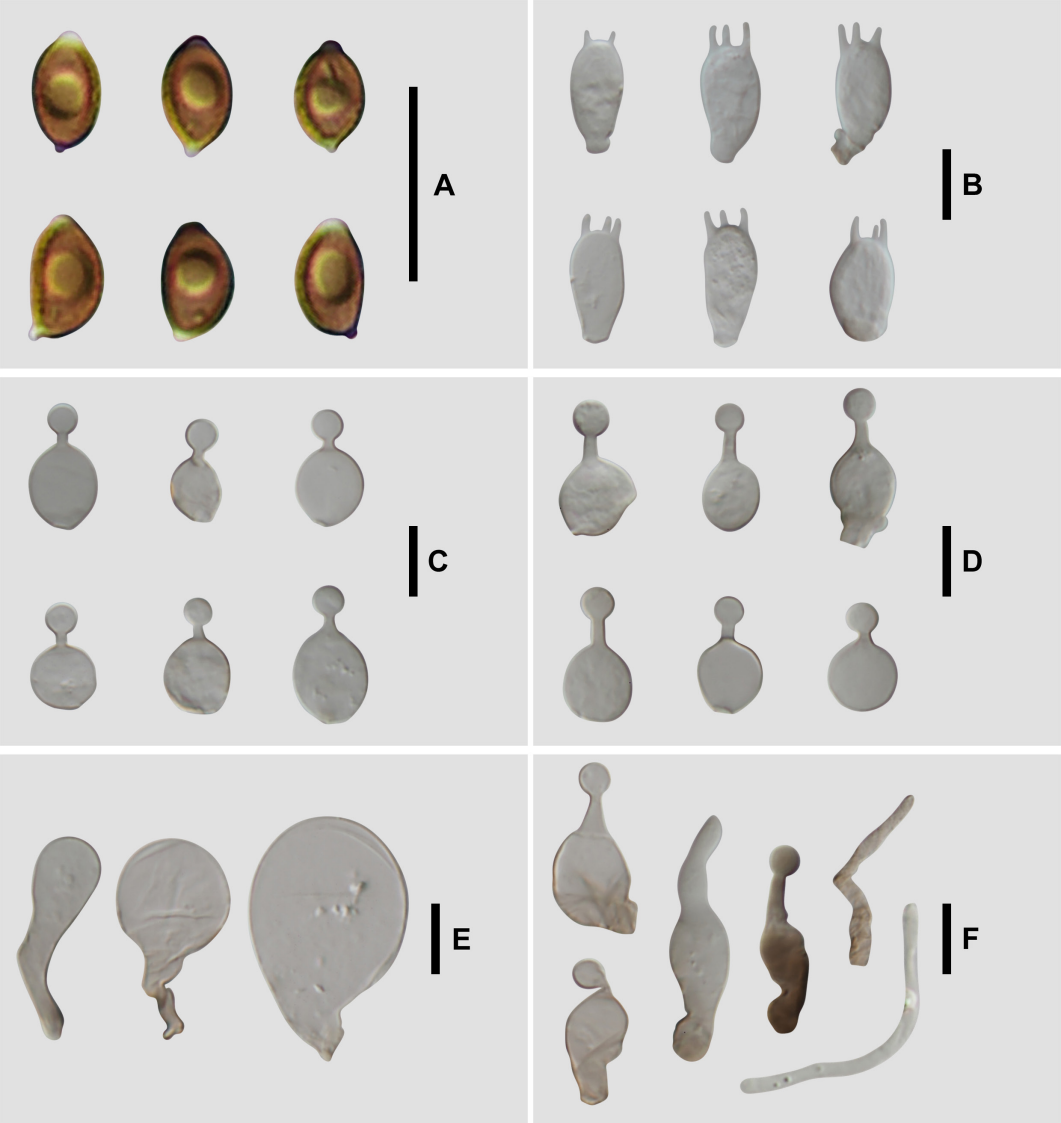
Microscopic structure images of *C. arenicola* (FJAU78830). **A**. Basidiospores; **B**. Basidia; **C**. Cheilocystidia; **D**. Caulocystidia; **E**. Pileipellis; **F**. Pileocystidia. Scale bars: 10 μm (**A–F**).

**Figure 8.**
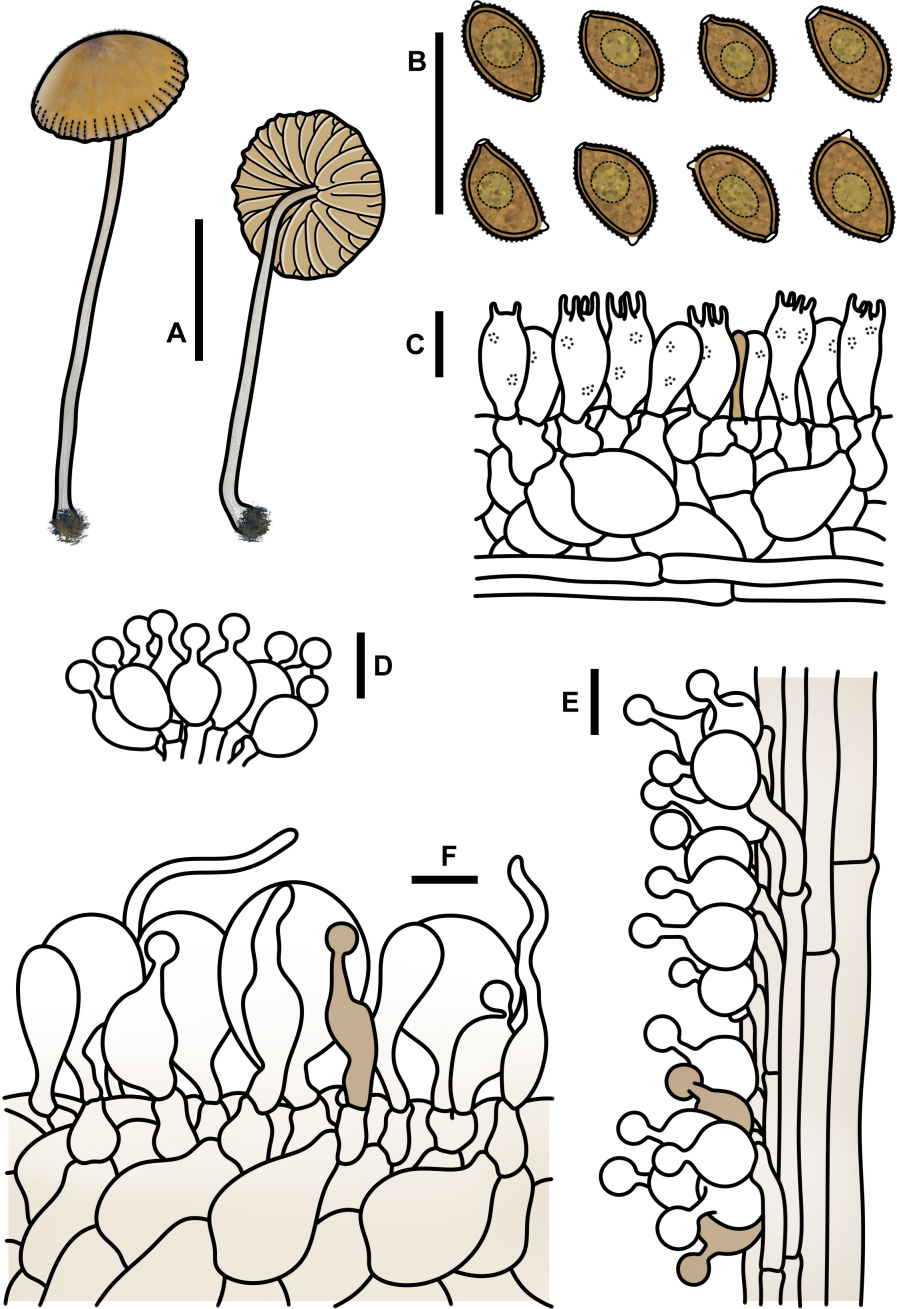
*Conocybe arenicola* (FJAU78830). **A**. Basidiomata; **B**. Basidiospores in KOH; **C**. Hymenium and subhymenium; **D**. Cheilocystidia; **E**. Stipitipellis; **F**. Pileipellis. Scale bars: 0.5 cm (**A**); 10 μm (**B–F**).

Figs 2F–J, 3C, 3D, 4C, 4D, 7, 8

##### Etymology.

“arenicola” refers to species that grow in sandy habitats.

##### Holotypus.

China • Jilin Province, Songyuan City, Hadashan Town, Songhua River bank, 27 August 2023, 45°8'13"N, 125°15'22"E, alt. 186 m, Han-bing Song SH2308114 (FJAU78830).

##### Diagnosis.

The main characteristics of this species include a pubescent pileus, papillate germ pore, lecythiform cheilocystidia, caulocystidia, and pileocystidia, and pileipellis with hair-like elements. It shows a weakly positive reaction with ammonia solution and grows in sandy soil. This combination of features distinguishes it from other species within *Conocybe* sect. *Ochromarasmius*.

##### Description.

Basidiomata mycenoid. Pileus diameter 0.3–1 cm, initially convex, later plano-convex to applanate, subumbonate, margin straight, undulate. Center sepia brown (RAL 8014) to mahogany brown (RAL 8016), chestnut brown (RAL 8003) to brown beige (RAL 1011), margin light ivory (RAL 1015) to ivory (RAL 1014), sand yellow (RAL 1002) to ochre brown (RAL 8001). Hygrophanous, pubescent, striations distinct, extending halfway to center. Context thin, color same as pileus, odor absent, taste absent. Lamellae adnexed to narrowly adnate, ventricose, distant, unequal length, beige (RAL 1001) to sand yellow (RAL 1002), brown beige (RAL 1011) to ochre brown (RAL 8001), margin even to slightly eroded. Stipe length 0.5–2 cm, thickness 0.5–1 mm, cylindrical, slightly transparent, ivory (RAL 1014) to brown beige (RAL 1011) or clay brown (RAL 8003). Surface pruinose or shortly pubescent (one specimen), longitudinally fibrillose-striate, base subbulbous.

##### Basidiospores.

(60/3/3) (5.2–)5.5–6.6(–6.9) × (3.2–)3.3–4.1(–4.3) μm, *Q* = (1.43–)1.46–1.83(–1.94), *Qm* = 1.63(±0.10), non-lentiform, ellipsoid to oblong, slightly amygdaliform, wall thick, ornamentation warty to pointed, faintly tuberculate-roughened, containing oil droplets, some germ pore papillate, approximately 1 μm diameter, in KOH ochre brown (RAL 8001) to clay brown (RAL 8003). Basidia 14–20(–21) × 7–9 μm, clavate, 4-spored, occasionally 2-spored, sterigmata 3–5 μm long, vacuolar inclusions present. Cheilocystidia (15–)16–20 × 7–11 μm, lecythiform, capitula 4–6 μm wide. Pleurocystidia absent. Caulocystidia (14–)15–21(–22) × (8–)9–12 μm, lecythiform, capitula 4–6 μm wide, capilliform elements occasionally observed (one specimen). Pileipellis epithelioid hymeniderm, cells 21–35(–36) × 17–28(–32) μm, spheropedunculate to clavate, base with brown beige (RAL 1011) pigment. Pileocystidia (20–)21–33(–35) × (4–)6–10(–12) μm, predominantly lecythiform, capitula 3–5 μm wide, some pigment-filled, sparsely scattered lageniform to long-necked lageniform or capilliform elements to 80 μm. Clamp connections present on all structures. Reaction with ammonia solution weakly positive, forming crystalline deposits.

##### Habitat.

It grows scattered in sandy grasslands during summer.

##### Known distribution.

Jilin Province, China.

##### Additional specimens measured.

China • Jilin Province, Songyuan City, Hadashan Town, Songhua River bank, 27 August 2023, 45°8'13"N, 125°15'22"E, alt. 186 m, Han-bing Song, S23082721 (FJAU78831); China • Jilin Province, Songyuan City, Fuyu City, Songhua River bank, 27 August 2023, 45°37'31"N, 121°54'56"E, alt. 259 m, Han-bing Song, S23082704 (FJAU78832); China • Inner Mongolia Autonomous Region, Tongliao City, Xar Moron River Park, 19 July 2023, 43°37'52"N, 122°15'35"E, alt. 259 m, Xian-yan Zhou, y2371916 (FJAU78833).

##### Notes.

Within subsection *Dumetorae*, this species is distinguished from *Conocybe dumetorum* and *C. dumetorum* var. *phaeoleiospora* by the latter two exhibiting a negative reaction to ammonia and having basidiospores with a non-papillate germ pore (Hausknecht 1995). While both this species and *C. dumetorum* var. *laricina* share a papillate basidiospore germ pore, the latter is separated by its negative ammonia reaction, slightly rugose, initially pruinose pileus, and caulocystidia that consist of a mixture of cylindrical to clavate elements (Hausknecht 1995). It differs from *C. abjecta*, which has slightly larger basidiospores (up to 8.5 μm long), mixed lecythiform and non-lecythiform caulocystidia, and lacks pileocystidia (Hausknecht 2003). It differs from *C. spinulosa* in having non-lecythiform caulocystidia and occurring on rotten hardwood in tropical rainforests (Hausknecht and Krisai-Greilhuber 1998). This species is distinguished from *C. stictospora* by the latter possessing mixed lecythiform and non-lecythiform caulocystidia and lacking pileocystidia (Singer 1989). It differs from *C. missionum*, which has slightly larger, somewhat angular basidiospores and a type locality in Argentina (Singer and Digilio 1951). The species is separated from *C. horakii*, which is characterized by its caulocystidia that comprise approximately 25% lecythiform and 75% non-lecythiform elements, and its growth on wood of *N. fusca* in New Zealand (Watling and Taylor 1987). It is distinguished from *C. mandshurica* by the latter’s obtusely conical to conical-convex pileus and larger basidiospores (Malysheva 2012a). This species differs from *C. incerta*, which has lecythiform cystidia (including cheilocystidia, caulocystidia, and pileocystidia) with a larger capitulum diameter (Malysheva 2017). Phylogenetically, this species is closely related to *C. subfusispora*, with the two forming a sister group relationship. However, they are distinguished by the latter having slightly larger, subfusiform basidiospores.

#### Conocybe subfusispora

Taxon classification

Fungi

AgaricalesBolbitiaceae

H.B. Song & Z.L. Luo sp. nov.

0BE6A06F-F62B-5144-99C9-57DAA7D8F77C

861725

**Figure 9.**
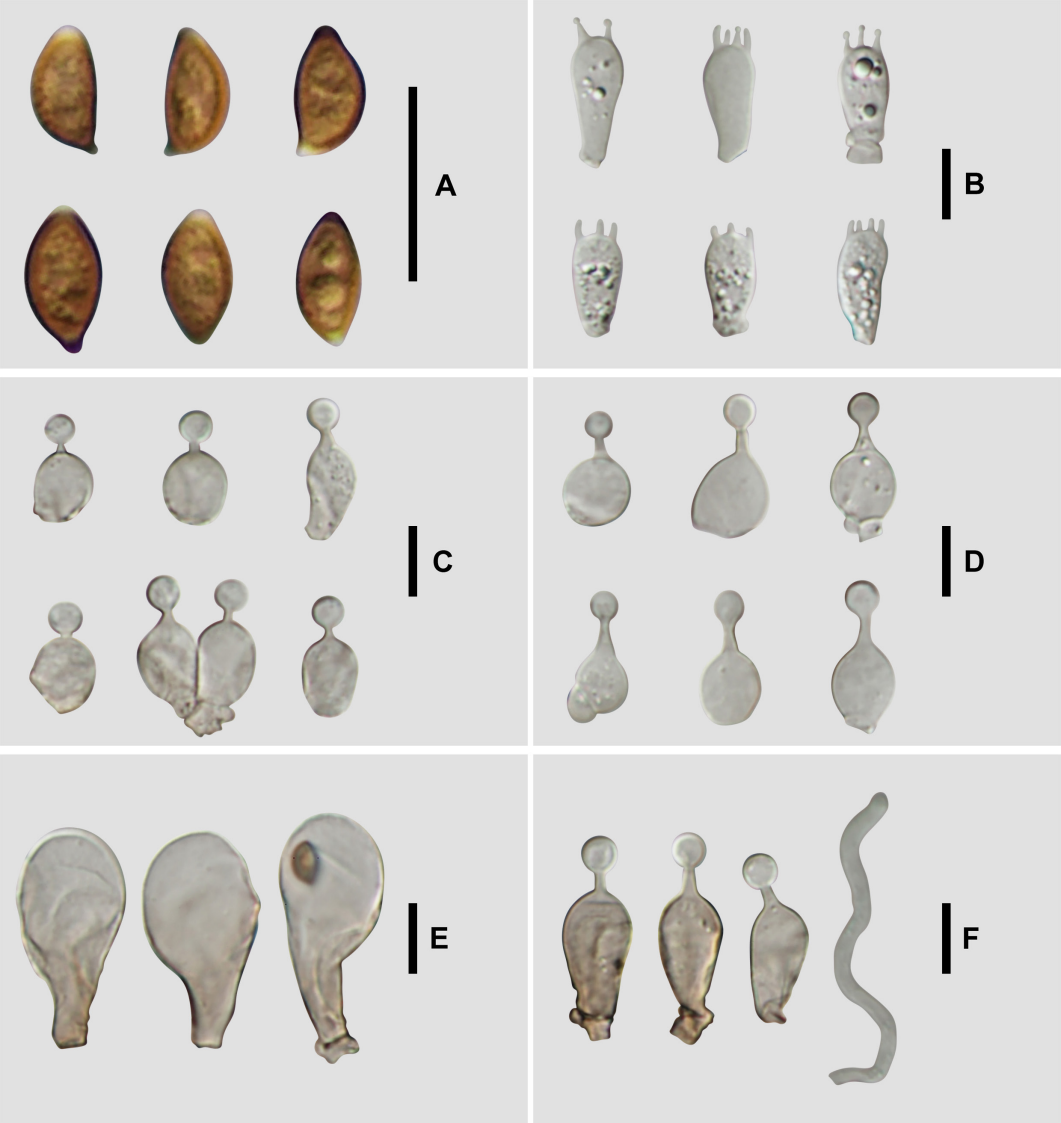
Microscopic structure images of *C. subfusispora* (FJAU78834). **A**. Basidiospores; **B**. Basidia; **C**. Cheilocystidia; **D**. Caulocystidia; **E**. Pileipellis; **F**. Pileocystidia. Scale bars: 10 μm (**A–F**).

**Figure 10.**
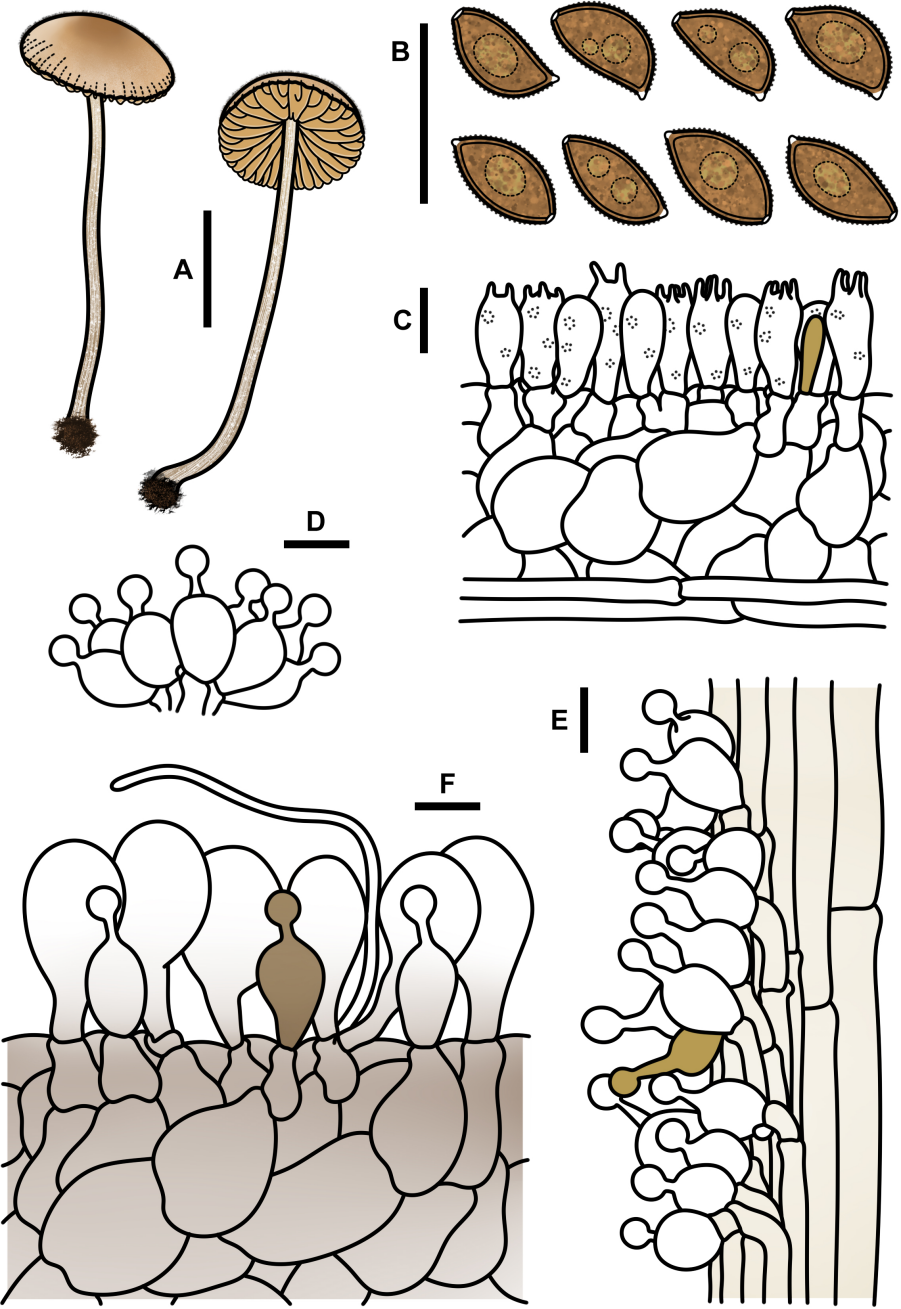
*Conocybe subfusispora* (FJAU78834). **A**. Basidiomata; **B**. Basidiospores in KOH; **C**. Hymenium and subhymenium; **D**. Cheilocystidia; **E**. Stipitipellis; **F**. Pileipellis. Scale bars: 0.5 cm (**A**); 10 μm (**B–F**).

Figs 2K–P, 3E, 3F, 4E, 4F, 9, 10

##### Etymology.

“subfusispora” refers to basidiospores that are subfusiform in frontal view.

##### Holotypus.

China • Jilin Province, Tonghua City, Ji’an City, Yushan Park, 26 July 2025, 41°08'20"N, 126°10'39"E, alt. 247 m, Han-bing Song, S25072601 (FJAU78834).

##### Diagnosis.

The main characteristics of this species are a pubescent pileus, subfusiform basidiospores with a papillate germ pore, and a pileipellis intermixed with capilliform elements. Among these, the subfusiform basidiospores can distinguish it from most species in *Conocybe* sect. *Ochromarasmius*.

##### Description.

Basidiomata mycenoid. Pileus diameter 0.3–1 cm, initially convex to plano-convex, later applanate to plano-concave, margin straight, undulate. Center ochre brown (RAL 8001) to signal brown (RAL 8002), fawn brown (RAL 8007), margin saffron yellow (RAL 1017), brown beige (RAL 1011) to beige (RAL 1001). Hygrophanous, smooth, pubescent, moist condition distinctly striate, striations extending to center. Slight drying making striations less pronounced. Context thin, brown beige (RAL 1011) to beige (RAL 1001), odor absent, taste absent. Lamellae adnexed to narrowly adnate, ventricose, distant, unequal length, beige (RAL 1001) to ivory (RAL 1014), ochre brown (RAL 8001), margin even to slightly eroded. Stipe length 1–3 cm, thickness 0.5–1 mm, cylindrical, slightly transparent, ivory (RAL 1014) to sand yellow (RAL 1002), brown beige (RAL 1011), ochre brown (RAL 8001) to signal brown (RAL 8002), surface pruinose, longitudinally fibrillose-striate, base subbulbous.

##### Basidiospores.

(60/3/3) (6.2–)6.3–7.6(–7.8) × (3.4–)3.5–4(–4.3) μm, *Q* = (1.67–)1.7–1.97(–2.1), *Qm* = 1.85(±0.09), not lentiform, oblong, partly subcylindrical, front view subfusiform, side view slightly amygdaliform, wall thick, ornamentation from warty to pointed, faintly tuberculate-roughened, containing oil droplets, germ pore papilla-shaped, approximately 1 μm in diameter, basidiospores in KOH clay brown (RAL 8003) to ochre brown (RAL 8001). Basidia (13–)15–19(–20) × (6–)7–9 μm, clavate, 4-spored, occasionally 2-spored, sterigmata 3–5 μm long, vacuolar contents present within basidia. Cheilocystidia 16–22 × 7–10 μm, lecythiform, capitula 3–5 μm wide. Pleurocystidia absent. Caulocystidia (15–)16–24(–26) × (7–)8–13 μm, lecythiform, capitula 4–6 μm wide. Pileipellis epithelioid hymeniderm, cells (16–)18–32 × (11–)12–19(–21) μm, spheropedunculate and clavate, base with signal brown (RAL 8002) pigment. Pileocystidia (18–)22–35(–37) × (8–)9–13(–14) μm, lecythiform, capitula 4–6 μm wide, some pigment-filled, intermixed capilliform elements to 70 μm. Clamp connections present on all structures. Reaction with ammonia negative.

##### Habitat.

It occurs scattered in broad-leaved forest grasslands and the humus layer during summer.

##### Known distribution.

Jilin Province, China.

##### Additional specimens measured.

China • Jilin Province, Tonghua City, Ji’an City, Yushan Park, 24 July 2025, 41°07'55"N, 126°10'42"E, alt. 213 m, Han-bing Song, S25072401 (FJAU78835), S25072402 (FJAU78836), S25072403 (FJAU78837); China • Jilin Province, Tonghua City, Ji’an City, Yushan Park, 26 July 2025, 41°08'20"N, 126°10'39"E, alt. 247 m, Han-bing Song, S25072602 (FJAU78838).

##### Notes.

Within subsection *Dumetorae*, this species is distinguished from *Conocybe dumetorum*, *C. dumetorum* var. *phaeoleiospora*, and *C. stictospora* by its basidiospore size and the papillate form of its germ pore (Hausknecht 1995; Singer 1989). While both this species and *C. dumetorum* var. *laricina* possess a papillate germ pore on their basidiospores, the latter differs in having a slightly rugose, initially pruinose pileus, slightly smaller basidiospores, and caulocystidia composed of a mixture of cylindrical to clavate elements (Hausknecht 1995). It differs from *C. abjecta*, which has caulocystidia of mixed lecythiform and non-lecythiform types and lacks pileocystidia (Hausknecht 2003). It is distinguished from *C. spinulosa* by the latter’s non-lecythiform caulocystidia and its occurrence on rotten hardwood in tropical rainforests (Hausknecht and Krisai-Greilhuber 1998). This species can be separated from *C. missionum*, which has slightly angular basidiospores and a type locality in Argentina (Singer and Digilio 1951). It is differentiated from *C. horakii*, whose caulocystidia consist of approximately 25% lecythiform and 75% non-lecythiform elements and which grows on wood of *N. fusca* in New Zealand (Watling and Taylor 1987). This species is distinct from *C. mandshurica*, which has an obtusely conical to conical-convex pileus and basidiospores lacking a papillate germ pore (Malysheva 2012a). It also differs from *C. incerta*, the latter having lecythiform cystidia (including cheilocystidia, caulocystidia, and pileocystidia) with a larger capitulum diameter (Malysheva 2017). Phylogenetically, this species forms an independent evolutionary clade. It is a sister to *C. arenicola* and is clearly distinct from all closely related species.

### Key to Species of *Conocybe* sect. *Ochromarasmius* subsect. *Dumetorae*

**Table:** 

1	Caulocystidia lecythiform	**2**
–	Caulocystidia non-lecythiform or mixed type	**10**
2	Germ pore papillate	**3**
–	Germ pore non-papillate	**6**
3	Basidiospores front view amygdaliform	**4**
–	Basidiospores front view not amygdaliform	**5**
4	Growing in sandy soil	***Conocybe arenicola***
–	Growing in humus-rich soil	***C. dumetorum* var. *laricina***
5	Basidiospores slightly angular	***C. missionum***
–	Basidiospores subfusiform	***C. subfusispora***
6	Pileus glabrous	**7**
–	Pileus pubescent	**8**
7	Pileus center nearly black-brown	***C. dumetorum* var. *phaeoleiospora***
–	Pileus center copper brown	***C. verrucosa***
8	Basidiospores greater than 7 μm	***C. mandshurica***
–	Basidiospores less than 7 μm	**9**
9	Cystidia capitulum diameter up to 11 μm	***C. incerta***
–	Cystidia capitulum diameter less than 7 μm	***C. dumetorum* var. dumetorum**
10	Pileocystidia absent	**11**
–	Pileocystidia present	**12**
11	Basidiospores less than 7.2 μm	***C. stictospora***
–	Basidiospores up to 7–8.5 μm	***C. abjecta***
12	Caulocystidia non-lecythiform	***C. spinulosa***
–	Caulocystidia mixed lecythiform and non-lecythiform	***C. horakii***

## Discussion

Based on the phylogenetic framework established by Tóth et al. (2013) and Song and Bau (2025), the phylogeny of section *Ochromarasmius* was reconstructed using a concatenated dataset of ITS, nrLSU, and *tef*1-α sequences. The analysis shows that *Conocybe* sect. *Ochromarasmius* subsect. *Dumetorae* forms a highly supported monophyletic clade (1/82), and its morphological characteristics are consistent with its phylogenetic position.

Regarding subsection *Dumetorae*, subsect. *Subdumetorae* is treated as a synonym. The rationale is as follows:

Based on morphology, Malysheva (2012a) considered the presence of hair-like elements in the pileipellis to be the key diagnostic feature for subsection *Subdumetorae*. However, such structures are also present in the pileipellis of *C. dumetorum*, the type species of subsect. *Dumetorae* (Hausknecht 1995). Furthermore, other members of subsect. *Dumetorae*, such as *C. arenicola* and *C. subfusispora*, also possess these hair-like elements in their pileipellis. This indicates that the defining characteristic of subsect. *Subdumetorae* is not exclusive and falls within the morphological range of subsect. *Dumetorae*. Additionally, within this group, the macroscopically observed pubescent appearance of the pileus typically correlates with the presence of hair-like elements in the pileipellis when viewed microscopically. As the pileocystidia in these taxa are relatively short, there is a consistent link between their macroscopic and microscopic features.

Phylogenetic analysis indicates that *Conocybe mandshurica* (the type species of subsect. *Subdumetorae*) and *C. dumetorum* (the type species of subsect. *Dumetorae*) form a highly supported clade (0.95/95) as sister groups, with their phylogenetic positions located within the monophyletic branch of subsect. *Dumetorae*. In summary, based on the congruent evidence from morphology and phylogeny, subsect. *Subdumetorae* should not be maintained as an independent subsection and is therefore treated as a synonym of subsect. *Dumetorae*.

Furthermore, within subsection *Dumetorae*, *Conocybe martiana* (Berk. & M.A. Curtis) Singer and *C. confundens* should be excluded from this subsection; indeed, they should not be retained within sect. *Ochromarasmius* at all. As concluded by Hausknecht and Krisai-Greilhuber (1998), *C. martiana* belongs to *Galerina* Earle. Meanwhile, *C. confundens* does not cluster phylogenetically with subsect. *Dumetorae*, and its basidiospore ornamentation is notably shallower than is typical for most species in sect. *Ochromarasmius* (Malysheva 2017). This situation parallels cases in other groups, such as that reported by Song et al. (2026) for *Conobolbitina* sect. *Lignicola* and sect. *Verrucisporae*, where differences in basidiospore ornamentation depth correspond to distinct phylogenetic lineages. Consequently, *C. confundens* is excluded from subsect. *Dumetorae*. Given that its morphological characteristics also do not align with those of other subsections within sect. *Ochromarasmius*, its removal from the section entirely is further proposed.

Currently, subsection *Dumetorae* includes 11 species: *Conocybe dumetorum*, *C. abjecta*, *C. spinulosa*, *C. stictospora*, *C. missionum*, *C. horakii*, *C. mandshurica*, *C. incerta*, *C. verrucosa*, *C. arenicola*, and *C. subfusispora*. An interesting phenomenon is observed among them. For those species with available sequence data, the ones forming a monophyletic clade all possess predominantly lecythiform caulocystidia. This group includes *C. dumetorum*, *C. mandshurica*, *C. incerta*, *C. verrucosa*, *C. arenicola*, and *C. subfusispora*. Among the remaining species for which sequence data are lacking, only *C. missionum* is described in its protologue as having exclusively lecythiform caulocystidia. Therefore, based on this morphological correlation, it is inferred that *C. missionum* also belongs to this clade and, by extension, has a phylogenetic position within subsect. *Dumetorae*.

The other four species (*Conocybe abjecta*, *C. spinulosa*, *C. stictospora*, and *C. horakii*) are characterized by non-lecythiform or mixed caulocystidia and currently lack molecular sequence data. Considering the proposed evolutionary trend within *Pholiotina* s.l. from non-lecythiform to lecythiform caulocystidia (Song and Bau 2024), a similar pathway may exist within sect. *Ochromarasmius*. If this hypothesis is correct, a further subdivision of subsect. *Dumetorae* might be warranted, which could necessitate the establishment of new subsections to accommodate some of its constituent species.

Subsection *Juruensis* contains only the single species, *Conocybe juruensis*. As the type species of sect. *Ochromarasmius*, it has caulocystidia of mixed lecythiform and non-lecythiform types and a pileipellis composed of spheropedunculate cells with distinct rostrate projections (Hausknecht and Krisai-Greilhuber 2006). The presence of rostrate spheropedunculate pileipellis cells might be diagnostic only at the species level and is not a stable character for delimiting higher taxonomic ranks. For instance, the monotypic sect. *Obscurae* Hauskn. & Krisai in *Conocybe* was established based on this feature; however, its type species, *C. obscura* Watling, has smooth basidiospores and non-lecythiform caulocystidia (Watling 1973; Hausknecht and Krisai-Greilhuber 2006). Similarly, the newly described species *C. rubrocyanea* T. Bau & H.B. Song (Song and Bau 2025) also exhibits rostrate spheropedunculate pileipellis cells, along with smooth basidiospores and non-lecythiform caulocystidia, yet it is placed within sect. *Pilosellae* Singer. Since molecular sequences are unavailable for both *C. juruensis* and *C. obscura*, the relative taxonomic importance of basidiospore ornamentation versus caulocystidia type cannot be conclusively evaluated at present. However, based on available evidence, caulocystidia morphology likely has greater systematic significance than the presence of rostrate projections on the pileipellis cells.

Subsections *Pleurocystidiatae* and *Subleiosporae* are both monotypic, containing only their respective type species, *Conocybe radicata* and *C. subleiospora*. Although both species possess pseudorhizas, they differ in that *C. radicata* has lecythiform pleurocystidia, whereas the caulocystidia in both are a mixture of lecythiform and non-lecythiform forms (Hausknecht 2009). Based on the presence or absence of pleurocystidia, Hausknecht placed them in separate subsections (Hausknecht 2009). This treatment is accepted here, although the taxonomic significance of pleurocystidia as a decisive character in *Conocybe* remains uncertain; it is known only that species possessing this structure are rarely encountered. However, within the genus *Conocybe*, rare diagnostic features are often informative only at the species level. If this principle holds, both *C. radicata* and *C. subleiospora* might belong in subsect. *Pleurocystidiatae*, rendering subsect. *Subleiosporae* synonymous. Alternatively, if pseudorhiza holds higher taxonomic priority than basidiospore ornamentation, both species should be classified within a group defined by pseudorhizas. However, considering species in sect. *Pilosellae*, such as *C. incarnata* (Jul. Schäff.) Hauskn. & Arnolds and *C. rufostipes* T. Bau & H.B. Song, which also have pseudorhizas but are not placed in such a pseudorhiza-defined group, this alternative seems less likely (Arnolds and Hausknecht 2003; Song and Bau 2023).

The primary contributions of this study are to clarify the distribution of *Conocybe* section *Ochromarasmius* subsection *Dumetorae* in China and elucidate the phylogenetic position of subsect. *Dumetorae*, and propose possible phylogenetic relationships among the remaining subsections and species within sect. *Ochromarasmius*. However, it remains uncertain whether sect. *Ochromarasmius* constitutes a monophyletic group. Future studies should obtain molecular sequences of the type species from each monotypic subsection under this section to validate the hypotheses discussed above.

## Supplementary Material

XML Treatment for Conocybe

XML Treatment for Conocybe

XML Treatment for Conocybe verrucosa

XML Treatment for Conocybe arenicola

XML Treatment for Conocybe subfusispora

## Additional information

### Artificial Intelligence (AI) use

The authors accept full responsibility for the content of the manuscript, including the disclosure of any use of AI.

No AI tools were used in the preparation of this manuscript.

### Author ORCIDs

J. Tao https://orcid.org/0009-0008-2897-6941

Y. Sheng https://orcid.org/0009-0007-8445-2715

J.-c. Zhao https://orcid.org/0009-0000-3043-9573

Z.-d. Xiong https://orcid.org/0009-0000-5543-4672

S.-m. Tang https://orcid.org/0000-0002-6174-7314

Z.-l. Luo https://orcid.org/0000-0001-7307-4885

H.-b. Song https://orcid.org/0000-0002-7440-5444

### Data availability

All the sequences have been deposited in GenBank (https://www.ncbi.nlm.nih.gov), and the new species names have been registered in MycoBank (https://www.mycobank.org). The data presented in this study are deposited in the Zenodo repository, accession number https://doi.org/10.5281/zenodo.18143088.
